# The Relationship Between EFL Teachers' Personality Traits, Communication Strategies, and Work Engagement

**DOI:** 10.3389/fpsyg.2022.855837

**Published:** 2022-03-07

**Authors:** Kunmin Ding, Lili Zhu, Xiujing Yan

**Affiliations:** Department of Foreign Language, Mudanjiang Medical University, Mudanjiang, China

**Keywords:** communication strategies, personality traits, work engagement, EFL teachers, language teaching

## Abstract

This review strives to shed light on the related studies on the relationship between English as a Foreign Language (EFL) teachers' personality traits, communication strategies, and their work engagement. The positive correlation between teachers' personality traits and work engagement has been confirmed in the review of the literature. Furthermore, studies have proved the relationship between teachers' communication strategies and personality traits. No studies have been done on the direct relationship between teachers' communication strategies and work engagement. However, the studies showed that some factors, such as teacher self-efficacy and willingness to communicate, can mediate the relationship between teachers' communication strategies and work engagement. To improve the language teaching quality, the pedagogical implications are explained in the end. Some suggestions for further research are provided to expand the literature about teachers' communication strategies, work engagement, and personality traits.

## Introduction

Positive educational contexts are significant for learners' academic success and performance (Pianta and Hamre, 2009). There are some teacher-related factors that are critical in educational contexts, and important for language learners. The current study examined how instructors' personality types are associated with their communication strategies and work engagement. Costa et al. (1995) defined personality as “the relatively enduring style of thinking, feeling, and acting that characterizes an individual” (p. 124). It denotes individuals' mindsets in coping with numerous events in various social contexts (Talasbek et al., 2020). Clayson and Sheffet (2006) stated that individuals possess personality traits, as specialized dimensions of personality, which relatively indicate their behavior. Communication strategies have also been defined differently from various perspectives in L2 contexts (Rabab'ah, 2014). According to Corder (1978), communication strategy refers to “a systematic technique practiced by the speakers when faced with difficulty to express the intended meaning” (p. 73). Poulisse (1987) argued that communication strategies are regarded as plans that individuals use in coping with problems in communication. Schaufeli et al. (2002) pointed out that work engagement refers to “a positive, fulfilling, work-related state of mind that is characterized by dedication, absorption, and vigor” (p. 75). Hakanen et al. (2006) mentioned that feelings of eagerness, motivation, arrogance, and challenges are related to dedication. They maintained that absorption, as another aspect of work engagement, is related to the full concentration of work. They also described vigor as “high levels of energy and mental resilience while working, the willingness to invest effort in one's work, and persistence also in the face of difficulties” (p. 498). Few studies have been done on teachers' work engagement (e.g., Minghui et al., 2018; Perera et al., 2018a,b; Fu et al., 2020). This study attempts to have a review on the relationship between work engagement, communication strategy, and personality traits. This study is significant since language instructors can obtain a complete understanding of their use of communication strategies, personality types, and engagement.

## Major Concepts on the Spotlight

### Personality Traits

Costa and McCrae (1992) identified neuroticism, conscientiousness, extraversion, agreeableness, and openness to experience as Big Five personality traits that have drawn the attention of many psychologists. They argued that neurotic individuals usually have negative emotions, including humiliation, cynicism, and low confidence. They maintained that reliable people have high levels of trustworthiness, and they are strong-minded and accountable in their life. They also stated that extroverts have high levels of gregariousness and self-assuredness. The high agreeable individuals tend to be easygoing, trusting, and considerate. Finally, they asserted that openness to experience is typified by characteristics like broad-mindedness, resourcefulness, and individuality in making decisions. Few investigations have been conducted on teachers' personality types. Asanjarani et al. (2022) found out that individuals' psychoticism and neuroticism predict their goal orientations. Kim et al. (2019) investigated the relationship between teachers' personalities and burnout. Using Big Five Personality Domain, they found out that extroverts and conscientious individuals are less likely to be emotionally exhausted. Kim et al. (2018) argued that the teachers' personality traits are significantly correlated with their support and learner self-efficacy. MacIntyre et al. (2019) study revealed that teachers' personality traits predicted both their stress levels and psychological well-being. Using Big-Five Factor Markers, they argued that supportive teachers are likely to construct social relationships that protect stress. Moreover, their study showed that stress decreases instructors' level of sociability. Rajabi and Ghezelsefloo (2020) found out that teachers' self-compassion and personality traits are highly significantly related to psychological well-being. Khalilzadeh and Khodi (2021) also found out that extravert teachers negatively influences the motivation of language learners. In other words, active and gregarious teachers cause learners not to engage in the classroom and learn the language.

### Communication Strategies

Ya-ni (2007) stated that non-native individuals' employment of communication strategies is usually influenced by their insufficient language knowledge. He noted that communication strategies can contribute individuals to using their communication in L2. Numerous studies have been done on learners' communication strategies (Habók and Magyar, 2018; e.g., Demir et al., 2018; Vafadar et al., 2020; Zheng et al., 2021a,b). Few studies have explored teachers' communication strategies in educational contexts. Yaghoubi-Notash and Karafkan (2015) investigated the functions of EFL teachers' communication strategies. Using Jamshidnejad's (2011) pattern about the roles of communication strategies, they found out that keeping the conversation flow is significantly different between instructors with various competency levels. Yaghoubi-Notash and Karafkan (2012) categorized elementary and advanced EFL teachers' communication strategies into L1/L3-based strategies, IL-based strategies, propositional reduction strategies, direct strategies, IL-based, and cooperative strategies. They found out that advanced-level EFL teachers significantly used propositional reduction strategies. Al-Gharaibeh and Al-jamal (2016) categorized teachers' communication strategies into comprehension- check, code-mixing, other-repetition, asking for repetition, hesitation devices, and guessing. They found out that “other repetition” is used more by EFL teachers, and they are unconscious in using communication strategies in educational contexts. Anani Sarab's (2004) study revealed that conversational modifications and lexical compensatory strategies are two frequently-used strategies that teachers employ in their talk. However, these strategies are not different between native and non-native ones.

### Work Engagement

According to Zhang and Yang (2021), teacher work engagement is significantly related to learner academic engagement in EFL contexts. They argued that dedicated and absorbed instructors can provide inspiring educational contexts in which learners tend to engage in the learning process. Han and Wang (2021) found out that instructors' work engagement and self-efficacy correlate significantly with their reflection in educational contexts. They argued that teachers with high confidence levels dedicate their time, expense, and vigor to their work and fervently do their job. Noughabi et al. (2020) found out that teacher work engagement and autonomy significantly correlate with teacher immunity. They justified their results by explaining resilience as a possible predictor of teacher immunity, and this factor can help teachers devote their time to coping with educational difficulties. Rusu and Colomeischi's (2020) study also revealed that teachers' engagement intervenes in the correlation between well-being and positive and negative emotions among teachers. Greenier et al. (2021) showed that teacher well-being and emotional regulator strategies significantly correlate with teacher engagement. They argued emotional regulation strategies used by teachers are effective for their involvement in doing educational tasks.

### The Relationship Between Personality Types and Work Engagement

The relation between personality types and work engagement has been discussed in a few studies. Work engagement is considered an essential element that influences job performance (Ismail Gulamali, 2017). Ongore (2014) stated that university instructors' personality traits are significantly correlated with their work engagement. His study revealed that extroverted, agreeable, and conscientious instructors have higher levels of cognitive and emotional engagement. He maintained that the affable instructors, seeking experience, are more inclined to have job engagement. He argued that teachers' openness to experience has changed teachers' working life. Nayyar et al. (2013) underscored the importance of “extraversion,” “agreeableness,” “conscientiousness,” and “openness to experience” in affecting teachers' work engagement. Rezaei et al. (2019) demonstrated that experienced teachers are inclined to have higher levels of ambiguity tolerance. They maintained that less ambiguity tolerant novice teachers are less engaged in classrooms. Li et al. (2017) study revealed that initiative EFL teachers are more engaged in their classes. They also found out that conscientious teachers have a tendency to be engaged in their classrooms. Perera et al. (2018a,b) investigated the relationship between teachers' personality types, job satisfaction, self-efficacy, and work engagement. They found out that excitable teachers have lower levels of work engagement. They argued that excitable EFL teachers have higher levels of extraversion related to the high levels of work engagement. However, these teachers have conscientiousness and higher neuroticism and agreeableness, which are harmful for work engagement. They also argued that excitable and compliant teachers are inclined to do the tasks in EFL educational contexts.

### The Relationship Communication Strategies and Personality Traits

Investigations have shown individuals' language learning strategies, and communication strategies are affected by multiple personality traits. Ehrman and Oxford (1990), in their study, revealed that extrovert teachers, compared to introverts, usually use effective strategies. However, introverted teachers widely use communication strategies in their talk. They also found out that intuitive teachers, compared to sensitive ones, widely employ communication strategies. Safranj and Gojkov-Raji (2019) found out that individuals with various types of personality traits have distinct inclinations in choosing learning strategies. Low conscientious learners frequently use communication strategies, while learners with great mental powers widely use metacognitive and cognitive strategies. They argued that less organized learners usually use communication strategies to prevail over the communication problems to carry on the flow of communication. However, Tarome (1977) mentioned that learners and teachers' personality traits influence their use of strategies in communication. Nikoopour and Amini Farsani (2011) specified EFL teachers' personality traits and their language learning strategies. They found out that extroverted and introverted teachers have significant differences in their language learning strategies. They stated that intuitive teachers use cognitive strategies, while judging teachers usually use communication strategies. Ahmadian and Yadgari (2011) showed that extroverted individuals, compared to introverts, significantly employ interactional strategies and transliteration. They argued that teachers' emotional features make extraverts widely use transliteration. Extraverts are interested in faster and less accurate methods, and they are inclined to talk thoughtlessly. Mutlu (2018) investigated the relationship between communication strategies and personality types. He found that personality types are not significantly correlated with learners' use of strategy types. Marpaung and Widyanotoro (2019) indicated that learners' oral strategies in communication are not affected by their personality traits. Farizah (2021) did not find any significant difference between teachers' communication strategies and their personality traits. Their study showed that introverted and extraverted teachers do not have a significant difference in their use of non-verbal strategies and reduction strategies. They argued that introverted and extroverted individuals try to have an efficient communication process through employing verbal strategies in communication to deal with gaps that they cope with while interacting with learners.

### The Relationship Between Communication Strategies and Work Engagement

Few studies have been done on the positive effect of teacher talk and their strategy use on learner engagement (e.g., Berry, 2006; Connolly et al., 2019; Chen et al., 2020). To our knowledge, no research has been done on the direct relationship between teachers' communication strategies and work engagement. However, self-efficacy, as a mediator, can play a role between these two constructs (Kusuma, 2019). Shirkhani and Mir Mohammad Meigouni (2020) found a significant relationship between communication strategies employed by teachers and their self-efficacy. Also, Kusuma (2019) found out that effective instructors tend to use strategies of communication. His study showed that teachers' oral communication strategies and self-efficacy predict their communicative competence in educational classrooms. However, Abbasi and Nosratinia (2018) found a significant relationship between self-efficacy and learners' communication strategies. They argued that learners' self-perceived image is one of the issues in developing EFL learners' speaking skill. On the other hand, it has been approved in a few studies that teachers' self-efficacy and their engagement in educational contexts are significant (Skaalvik and Skaalvik, 2014; Granziera and Perera, 2019). Skaalvik and Skaalvik's (2014) study indicated that teachers who are more engaged in the classrooms have higher self-efficacy and self-sufficiency. They argued that teacher self-efficacy verifies the perception of contextual opportunities for language learning and their work engagement. Granziera and Perera (2019) stated that teacher work engagement has a mutual relationship with teacher self-efficacy. They argued that “teachers' principles that they can perform certain teaching-related tasks may serve as a crucial internal resource for mobilizing attention and efforts in performing work-related tasks” (p. 8). Willingness to communicate, as another variable, can be considered important in the connection between teachers' communication strategies and work engagement (Yaraghi and Shafiee, 2018).

## Concluding Statements

Teachers' communication strategies, work engagement, and personality traits are important for the growth of instruction in educational contexts. This review inspected the relationship between teachers' communication strategies, personality traits, and work engagement. This review enriched our understanding of teachers' personality traits, work engagement, and their use of communication strategies. The literature review verified the significant and positive correlation between teachers' communication strategies and their personality types. Moreover, it has been proved that teachers' work engagement and communication strategies have a significant relationship with each other. This review enhances the educational knowledge of investigators who are interested in teachers' personality types and communication strategies. Considering the related studies on the role of learners' affective factors on teacher engagement, it can be mentioned that learners should be assisted to control, adjust, and regulate their emotions in language learning contexts to help teachers engage in classroom contexts. Language instructors are required to decrease apprehension, boredom, and anxiety sources among learners to improve work engagement. This review implicates that instructors can change their engagement both by employing different methods and regulating their outward feelings. Teachers should express their personality traits to supervisors and their colleagues. They should state their challenges and concerns about instructional and organizational problems, and educational contexts in order to keep on work engagement. They should raise their awareness of the relationship between communication strategies, work engagement, and their personality types. Therefore, they can devote their energy to develop their instructional abilities by pondering into their practices which increase their efficiency and participation in their work, and their use of effective communication strategies. In addition, educational supervisors, who observe instructors and gauge their engagement, can make use of the related studies through consideration of the instructors' personality traits. If teachers do not use communication strategies in educational contexts, the interaction between learners and teachers is affected which triggers teacher educators to consider this issue in practical aspects. This review recommends that teacher educators should have a positive view toward teachers, and they should provide well-organized and inspiring teaching methodologies which can construct their personality traits, and increase teachers' work engagement. Teacher trainers should concentrate on providing some strategies to enhance positive feelings and personality types to engage teachers in educational contexts. Furthermore, teachers' work engagement can be developed through poems, arts, and melodies, since these techniques are associated with work engagement (Croom, 2015). Given the effectiveness of personality traits on teachers' work engagement, teacher educators are required to build pre-and in-service language teachers' positive types of personality to deal with the problems and difficulties of teaching.

This review can also inspire school principals and policymakers to consider EFL teachers' personality traits. They are required to develop independent, sociable, and prepared educational contexts where EFL instructors can enhance their communication strategies, levels of work engagement, and improve their positive personality traits. They can organize and design curricula that reduce teacher boredom, apprehension, frustration, and shame, and increase their enjoyment for fostering teacher work engagement. They can also hold intervention programs and workshops to consider teachers' use of communication strategies. The schools and institutes' managers should provide EFL contexts supporting teachers' work engagement by offering authentic, joyful, and updated materials to teachers and learners.

This review has some suggestions for further research. Teachers' communication strategies emotions with different educational levels should be investigated. Investigations need to be done on teachers' communication strategies in numerous instructive, local, national, and cultural contexts. Studies should be done on the direct relationships between teachers' communication strategies and work engagement. Studies can also measure the effect teachers' emotional regulation on their use of communication strategies.

Regarding teachers' personality traits, the connection between EFL teachers' enjoyment, well-being, and their personality traits can be investigated for the future. More studies need to be done on the effect of instructors' personality types on their employment of methodologies should be considered. Moreover, future studies can highlight gender's effect on language teachers' personality types. Also, further research needs to be done on the effects of teachers' personality types on their working memory. Likewise, the effect of EFL teachers' personality types on their language skills should be meticulously regarded. The effect of learners' academic engagement on teachers' personality types can also be examined. Studies should investigate teachers' personality types in conventional and virtual educational contexts to brighten the influence of educational contexts on teachers' personality traits.

Regarding teachers' work engagement, further studies should examine the direct association between teachers' communication strategies and their work engagement. The relationship between teacher apprehension, boredom, shame, frustration, and work engagement should be studied. A detailed study is also needed to investigate the association between teacher resilience, as a component of positive psychology, and work engagement.

## Author Contributions

KD conceived of the presented idea and developed the theory. LZ took responsibility for writing. XY supervised the project. All authors contributed to the article and approved the submitted version.

## Funding

The research was supported by Construction of English Teaching Model Based on Blue-ink Cloud Class+BOPPPS in Application-oriented Universities, Teaching Reform Project of Higher Education in Heilongjiang Province, China (Grant No. SJGY20190707), Construction of Blended English Teaching Model Based on the Concept of Curriculum Ideology and Politics, and Teaching Reform Project of Higher Education in Heilongjiang Province, China (Grant No. SJGY20200767).

## Conflict of Interest

The authors declare that the research was conducted in the absence of any commercial or financial relationships that could be construed as a potential conflict of interest.

## Publisher's Note

All claims expressed in this article are solely those of the authors and do not necessarily represent those of their affiliated organizations, or those of the publisher, the editors and the reviewers. Any product that may be evaluated in this article, or claim that may be made by its manufacturer, is not guaranteed or endorsed by the publisher.
